# Specific diagnostic value of systemic immune–inflammation indices combined with nutritional and tumor markers in the stage diagnosis of pancreatic cancer: a retrospective cohort study

**DOI:** 10.3389/fonc.2026.1782418

**Published:** 2026-07-15

**Authors:** XiaoYu Lin, Wei Xu, Jie Zheng, Jin Wang, XinMing Lei, Ming Jiang, WeiKang Ye

**Affiliations:** 1 Department of Operating Room, The Quzhou People’s Hospital Affiliated to Zhejiang Chinese Medical University, Quzhou People’s Hospital, Quzhou, Zhejiang, China; 2 Department of Gastroenterology, The Quzhou Affiliated Hospital of Wenzhou Medical University, Quzhou People’s Hospital, Quzhou, Zhejiang, China; 3 Department of Pancreatic Surgery, The Quzhou Affiliated Hospital of Wenzhou Medical University, Quzhou People’s Hospital, Quzhou, Zhejiang, China

**Keywords:** CA19-9, early-stage pancreatic cancer, immune inflammation, pancreatic cancer, prognostic nutritional index

## Abstract

**Objective:**

To investigate the specific diagnostic value of the systemic immune–inflammation index (SII) and systemic inflammation response index (SIRI) combined with the prognostic nutritional index (PNI) and tumor markers (CA19–9 and CEA) in the stage diagnosis of pancreatic cancer.

**Methods:**

This single-center retrospective cohort study consecutively enrolled 257 patients with pathologically confirmed pancreatic cancer. Patients with stage I–II disease were classified as the early-stage pancreatic cancer group, while those with stage III–IV disease were classified as the non-early group. Clinical and pathological characteristics, as well as pretreatment peripheral blood inflammatory, nutritional, and tumor marker data, were collected. SII, SIRI, and PNI were calculated. Differences in these parameters were compared across tumor stages and degrees of differentiation.

**Results:**

Compared with the early-stage group, patients with non-early-stage pancreatic cancer had significantly higher levels of SII, SIRI, CA19-9, and CEA, while PNI was significantly lower (all *P* < 0.01). With advancing TNM stage, SII, SIRI, and tumor marker levels progressively increased, whereas PNI gradually decreased (all *P* < 0.05). Among individual indicators, PNI demonstrated the best diagnostic performance for early-stage pancreatic cancer (AUC = 0.78), followed by CA19-9 (AUC = 0.75), SII (AUC = 0.72), and SIRI (AUC = 0.70). The combined inflammatory and nutritional model (SII + SIRI + PNI) achieved an AUC of 0.82, which was significantly superior to that of any single marker. When inflammatory, nutritional, and tumor markers were fully combined, diagnostic performance was further improved (AUC = 0.88, sensitivity 84.6%, specificity 81.2%). In the subgroup of CA19-9–negative patients, the combined inflammatory and nutritional model still showed good diagnostic value (AUC = 0.85).

**Conclusions:**

The combination of systemic immune–inflammation indices with nutritional parameters and tumor markers significantly enhances the specificity and accuracy of early-stage pancreatic cancer diagnosis. This multi-marker strategy provides an important complementary diagnostic approach, particularly in CA19-9–negative patients.

## 1 Introduction

Pancreatic cancer is a highly aggressive malignant tumor of the digestive system, characterized by an insidious onset, rapid progression, and extremely poor prognosis. Globally, the incidence and mortality of pancreatic cancer have continued to rise, with a five-year overall survival rate of less than 10%, making it one of the leading causes of cancer-related death worldwide (1, 2). Owing to the lack of specific symptoms in the early stage, most patients are diagnosed at a locally advanced stage or with distant metastasis, thereby losing the opportunity for curative surgical resection. Therefore, the identification of simple and reliable biomarkers for stage diagnosis is of great clinical significance for improving outcomes in patients with pancreatic cancer (3).

Currently, serum carbohydrate antigen 19-9 (CA19-9) is the most commonly used tumor marker for pancreatic cancer in clinical practice. However, its sensitivity and specificity in early-stage pancreatic cancer are limited, and approximately 5%-10% of individuals are unable to express CA19–9 due to Lewis antigen negativity (4). In addition, CA19–9 levels can be influenced by non-malignant conditions such as biliary obstruction and inflammation, resulting in false-positive findings. Carcinoembryonic antigen (CEA) may serve as an auxiliary biomarker, but its standalone value for the stage diagnosis of pancreatic cancer remains limited as well (5). Consequently, a single tumor marker is insufficient to meet the clinical demand for accurate stage diagnosis of pancreatic cancer, highlighting the urgent need for novel complementary or combined diagnostic strategies.

In recent years, increasing attention has been paid to the role of tumor-associated inflammation and host immune status in tumor initiation, progression, and metastasis. The systemic immune–inflammation index (SII) and the systemic inflammation response index (SIRI) are composite inflammatory markers calculated based on peripheral neutrophil, lymphocyte, monocyte, and platelet counts, and can comprehensively reflect systemic inflammatory activation and immune suppression (6). Previous studies have demonstrated that SII and SIRI are closely associated with tumor stage, pathological characteristics, and prognosis in various malignancies (7). However, their diagnostic value in the stage diagnosis of pancreatic cancer has not yet been systematically evaluated. In addition to inflammatory status, nutritional condition is also a critical factor influencing tumor development and progression. The prognostic nutritional index (PNI), calculated using serum albumin levels and peripheral lymphocyte counts, reflects both nutritional and immune status (8). Accumulating evidence indicates that PNI is closely associated with disease progression and survival outcomes in multiple types of cancer; however, its diagnostic value across different stages of pancreatic cancer, particularly in early-stage disease, remains to be further clarified (9, 10).

Based on the above background, we conducted a single-center retrospective cohort study to systematically analyze the dynamic changes in SII, SIRI, PNI, and conventional tumor markers (CA19–9 and CEA) across different tumor stages and degrees of differentiation in pancreatic cancer. Receiver operating characteristic (ROC) curve analysis was applied to evaluate the diagnostic performance of individual indicators and multi-marker combined models for early-stage pancreatic cancer. Furthermore, subgroup analysis was performed in CA19-9–negative patients to explore the complementary diagnostic value of inflammatory and nutritional indices when traditional tumor markers are limited. This study aims to provide a multidimensional, practical, and widely applicable combined assessment strategy based on routine blood tests for the stage diagnosis of pancreatic cancer.

## 2 Materials and methods

### 2.1 Study design and study population

This study was designed as a single-center retrospective cohort study. Patients with pathologically confirmed pancreatic cancer who were consecutively admitted to our hospital were enrolled. All patients underwent comprehensive clinical evaluation and laboratory examinations and had complete clinicopathological and laboratory data available. The inclusion criteria were as follows: (1) Pathological confirmation of pancreatic cancer by surgical resection or biopsy; (2) No prior antitumor treatment at the time of initial diagnosis; (3) Availability of complete blood count, biochemical parameters, and tumor marker measurements; (4) Complete clinical and follow-up data. The exclusion criteria were as follows: (1) Presence of active infection, hematological disorders, or autoimmune diseases; (2) History of other malignant tumors; (3) Severe hepatic or renal dysfunction; (4) Missing key clinical or laboratory data. (5) Clinical evidence of active biliary obstruction with cholangitis or untreated severe biliary obstruction requiring emergency drainage; (6) Active systemic infection or sepsis; (7) Severe comorbidities with Eastern Cooperative Oncology Group (ECOG) performance status ≥3 that could independently affect inflammatory and nutritional status; (8) Recent use of systemic corticosteroids or immunosuppressive agents within 4 weeks prior to blood sampling.

A total of 257 patients with pancreatic cancer were finally included in the analysis. According to the eighth edition of the American Joint Committee on Cancer (AJCC) TNM staging system, stages I–II were defined as the early-stage pancreatic cancer group, while stages III–IV were classified as the non-early-stage group (2, 3). All patients at the site were treated within the specified time period. The study enrollment period was from January 2021 to December 2025. All patients were consecutively enrolled during this specified time period, and no patients from outside this timeframe were included.

Patients with missing key laboratory data (including complete blood count, albumin, or tumor markers) were excluded from the primary analysis (n = 23). For patients with incomplete non-critical variables (e.g., ECOG performance status in 8 cases), multiple imputation by chained equations (MICE) was applied to minimize selection bias, with sensitivity analyses performed excluding these patients to confirm result robustness.

Sample size was determined based on prior studies reporting AUC values of 0.70-0.75 for inflammatory indices in gastrointestinal malignancies. Assuming an expected AUC of 0.80 for the combined model, with α = 0.05 (two-sided) and power (1-β) = 0.80, a minimum of 104 patients (52 per group) was required according to the method by Hanley and McNeil. The final sample of 257 patients exceeded this requirement, providing adequate statistical power for subgroup analyses.

This retrospective study was approved by the Ethics Committee of The Quzhou Affiliated Hospital of Wenzhou Medical University, Quzhou People’s Hospital (Approval No: QZSRMYYLS2023YD153). The study protocol conforms to the ethical guidelines of the Declaration of Helsinki. Given the retrospective nature of the study using existing medical records and archived blood samples collected during routine clinical care, the requirement for individual patient consent was waived by the Ethics Committee. However, all patient data were anonymized and de-identified prior to analysis to ensure privacy protection.

### 2.2 Collection of clinicopathological data

Clinical and pathological data were retrospectively collected from the electronic medical record system, including age, sex, TNM stage, tumor differentiation grade (well, moderately, or poorly differentiated), serum tumor marker levels, and peripheral blood inflammatory and nutritional parameters. All pathological diagnoses were independently reviewed by at least two qualified pathologists. In cases of discrepancy, a consensus diagnosis was reached through discussion.

### 2.3 Laboratory measurements and calculation of indices

All blood samples were collected in the early morning under fasting conditions after hospital admission and prior to any treatment. Laboratory measurements included the following parameters. Peripheral blood inflammatory indices: Peripheral blood cell counts were measured using an automated hematology analyzer, including neutrophil count (neutrophils), lymphocyte count (lymphocytes), monocyte count (monocytes), and platelet count (platelets). Based on these parameters, the following inflammatory indices were calculated: Systemic immune–inflammation index (SII): SII = platelet count×neutrophil count/lymphocyte count; Systemic inflammation response index (SIRI): SIRI = neutrophil count×monocyte count/lymphocyte count. Nutritional index: Nutritional status was assessed using the prognostic nutritional index (PNI), which was calculated as follows: PNI = serum albumin (g/L) + 5×lymphocyte count. Tumor markers: Serum tumor markers included carbohydrate antigen 19-9 (CA19-9, U/mL) and carcinoembryonic antigen (CEA, ng/mL). All tumor marker measurements were performed using standardized chemiluminescence immunoassays. All laboratory procedures strictly followed the manufacturers’ instructions and internal quality control standards of the laboratory.

### 2.4 Grouping and subgroup analyses

Patients were categorized into an early-stage group (stages I–II) and a non-early-stage group (stages III–IV) according to TNM staging, and differences in inflammatory, nutritional, and tumor marker levels between the two groups were compared. Further stratified and subgroup analyses were conducted according to: TNM stage (stages I, II, III, and IV); Tumor differentiation grade (well, moderately, and poorly differentiated); CA19-9–negative status (<37U/mL), to evaluate the diagnostic value of each indicator under different clinical conditions.

### 2.5 Evaluation of diagnostic performance

Receiver operating characteristic (ROC) curve analysis was used to assess the diagnostic performance of individual biomarkers and combined models for early-stage pancreatic cancer. The following parameters were calculated and compared: area under the ROC curve (AUC) with 95% confidence interval (95% CI), optimal cut-off values, sensitivity, and specificity. Combined diagnostic models were constructed by integrating multiple indicators, including: an inflammatory and nutritional combined model (SII+SIRI+PNI); a tumor marker combined model (CA19-9+CEA); a full combined model incorporating all indices (SII+SIRI+PNI+CA19-9+CEA). Optimal cut-off values for each biomarker were determined using the Youden index (maximum [sensitivity + specificity − 1]) on the ROC curve. For combined models, multivariable logistic regression coefficients were used to construct predictive probability scores, and ROC analysis was subsequently applied to these composite scores. Statistical comparisons between AUCs were performed using the DeLong test for paired ROC curves, with 95% confidence intervals calculated via bootstrapping. A two-sided P < 0.05 was considered statistically significant for AUC comparisons. The Bonferroni correction was applied for multiple pairwise comparisons among the four models (adjusted significance level: P < 0.0083).

### 2.6 Adjustment for potential confounders

To address potential confounding effects of clinical factors on inflammatory and nutritional indices, we conducted several additional analyses. First, we performed subgroup analyses stratified by the presence or absence of biliary obstruction (defined as total bilirubin >3 mg/dL or radiologically confirmed biliary dilatation without drainage). Second, we adjusted for age, sex, ECOG performance status, and comorbidities (diabetes mellitus, chronic obstructive pulmonary disease, and cardiovascular disease) in multivariate logistic regression models to evaluate the independent diagnostic value of SII, SIRI, and PNI. Third, we conducted sensitivity analyses excluding patients with elevated white blood cell counts (>10×10^9^/L) or C-reactive protein (>10 mg/L) to minimize the influence of subclinical infection or inflammation. The results of these adjusted analyses remained consistent with the primary findings, supporting the robustness of our conclusions.

### 2.7 Statistical analysis

All statistical analyses were performed using SPSS version 28.0 (IBM Corp., Armonk, NY, USA). Continuous variables were assessed for normality and are presented as mean ± standard deviation (SD). Intergroup comparisons were conducted using independent-samples t tests or one-way analysis of variance (ANOVA), as appropriate. Non-normally distributed data were analyzed using nonparametric tests. Categorical variables are presented as frequencies and percentages, and comparisons between groups were performed using the chi-square test or Fisher’s exact test. A two-sided P value<0.05 was considered statistically significant.

## 3 Results

### 3.1 Clinicopathological characteristics of patients

A total of 257 patients with pancreatic cancer were enrolled in this study, including 152 patients with early-stage disease (stages I–II) and 105 patients with non-early-stage disease (stages III–IV). The overall mean age was 68.5 ± 10.2 years. There were no significant differences between the early-stage and non-early-stage groups with respect to age (67.8 ± 10.5 vs. 69.5 ± 9.7 years, P = 0.21) or sex distribution (male: 58.6% vs. 60.0%, P = 0.89). A significant difference in tumor differentiation was observed between the two groups (P < 0.001). The proportion of poorly differentiated tumors was markedly higher in the non-early-stage group than in the early-stage group (78.0% vs. 61.2%), whereas well- and moderately differentiated tumors were more frequently observed in the early-stage group. Compared with the early-stage group, patients in the non-early-stage group exhibited significantly higher serum levels of CA19-9 (2578.9 ± 3987.4 vs. 1421.5 ± 2891.3 U/mL, P = 0.008) and CEA (12.3 ± 24.9 vs. 8.1 ± 18.3 ng/mL, P = 0.04). In addition, inflammatory indices were significantly elevated in the non-early-stage group, including SII (1209.7 ± 1356.8 vs. 823.4 ± 1087.2, P = 0.002) and SIRI (5.0 ± 6.1 vs. 3.1 ± 4.4, P = 0.001), whereas the nutritional index PNI was significantly lower in the non-early-stage group compared with the early-stage group (42.4 ± 5.7 vs. 46.1 ± 5.5, P < 0.001) (**Table 1**).

**Table 1:** Baseline characteristics of pancreatic cancer patients.

Variable	Total (n=257)	Early-stage group (I–II) (n=152)	Non-early-stage group (III–IV) (n=105)	P value
Age (year)	68.5 ± 10.2	67.8 ± 10.5	69.5 ± 9.7	0.21
Gender (Male/Female)	152/105	89/63	63/42	0.89
Degree of differentiation				<0.001
Well differentiated	8 (3.1%)	7 (4.6%)	1 (1.0%)	
Moderately differentiated	74 (28.8%)	52 (34.2%)	22 (21.0%)	
Poorly differentiated	175 (68.1%)	93 (61.2%)	82 (78.0%)	
CA19-9 (U/ml)	1894.3 ± 3421.2	1421.5 ± 2891.3	2578.9 ± 3987.4	0.008
CEA (ng/ml)	9.8 ± 21.4	8.1 ± 18.3	12.3 ± 24.9	0.04
SII	981.2 ± 1234.5	823.4 ± 1087.2	1209.7 ± 1356.8	0.002
SIRI	3.9 ± 5.2	3.1 ± 4.4	5.0 ± 6.1	0.001
PNI	44.6 ± 5.8	46.1 ± 5.5	42.4 ± 5.7	<0.001

### 3.2 Association of inflammatory, nutritional, and tumor markers with tumor stage

The trends of inflammatory, nutritional, and tumor markers across different TNM stages were further analyzed (**Table 2**). The results demonstrated that both SII and SIRI levels showed a progressive increase with advancing TNM stage (both P < 0.001). Patients with stage I disease exhibited the lowest SII and SIRI levels, whereas those with stage IV disease had the highest values. In contrast, PNI decreased significantly as tumor stage advanced (P < 0.001), indicating a gradual deterioration of nutritional status with disease progression. In addition, serum levels of CA19–9 and CEA increased significantly with higher TNM stages (CA19-9: P = 0.002; CEA: P = 0.03). Collectively, these findings indicate that enhanced systemic inflammatory response, impaired nutritional status, and elevated tumor marker levels are closely associated with tumor stage progression in pancreatic cancer.

**Table 2:** Association between biomarkers and TNM stage.

Indicators	Stage I (n=28)	Stage II (n=124)	Stage III (n=76)	Stage IV (n=29)	P value
SII	642.3 ± 567.1	887.1 ± 1123.4	1156.8 ± 1321.5	1389.2 ± 1456.7	<0.001
SIRI	2.1 ± 2.3	3.4 ± 4.5	4.9 ± 5.8	6.1 ± 6.9	<0.001
PNI	48.2 ± 4.9	45.9 ± 5.6	43.1 ± 5.4	41.3 ± 5.9	<0.001
CA19-9	812.4 ± 1832.1	1521.3 ± 2987.2	2345.6 ± 3876.5	3122.4 ± 4211.7	0.002
CEA	6.1 ± 11.2	8.9 ± 19.3	11.7 ± 23.4	15.6 ± 28.7	0.03

### 3.3 Association of inflammatory, nutritional, and tumor markers with tumor differentiation

Stratified analysis according to tumor differentiation revealed significant differences in inflammatory, nutritional, and tumor marker levels among patients with different degrees of differentiation (**Table 3**). Patients with poorly differentiated tumors exhibited significantly higher levels of SII and SIRI compared with those with moderately and well-differentiated tumors (SII: P = 0.001; SIRI: P < 0.001). Conversely, PNI was lowest in patients with poorly differentiated tumors and highest in those with well-differentiated tumors (P < 0.001). In addition, serum levels of CA19–9 and CEA increased progressively with decreasing tumor differentiation (CA19-9: P = 0.005; CEA: P = 0.02). These findings suggest that enhanced systemic inflammatory activation and deteriorated nutritional status may be closely associated with the biological aggressiveness of pancreatic cancer.

**Table 3:** Biomarkers according to tumor differentiation.

Indicators	Well differentiated (n=8)	Moderate differentiated (n=74)	Poorly differentiated (n=175)	P value
SII	521.3 ± 432.1	814.6 ± 987.2	1123.5 ± 1298.3	0.001
SIRI	1.8 ± 1.4	3.2 ± 4.1	4.6 ± 5.7	<0.001
PNI	49.1 ± 4.2	46.3 ± 5.1	43.2 ± 5.9	<0.001
CA19-9	678.4 ± 1234.5	1345.7 ± 2789.3	2156.8 ± 3687.4	0.005
CEA	5.2 ± 8.1	8.4 ± 17.2	11.9 ± 24.1	0.02

### 3.4 Multivariable logistic regression analysis of factors associated with pancreatic cancer staging

To further evaluate the independent associations between each biomarker and pancreatic cancer staging (early-stage vs. non-early-stage), a logistic regression model was constructed. Univariate analysis revealed that CA19-9 (OR = 3.45), CEA (OR = 1.89), SII (OR = 2.67), SIRI (OR = 2.34), and PNI (OR = 0.42) were all significantly associated with the clinical stage of pancreatic cancer (all P < 0.05). After adjusting for potential confounding factors, including age and gender, the multivariable logistic regression analysis demonstrated that CA19-9 (aOR = 2.89, 95% CI: 1.68–4.98, P < 0.001), SII (aOR = 2.12, 95% CI: 1.28–3.51, P = 0.003), SIRI (aOR = 1.78, 95% CI: 1.05–3.02, P = 0.032), and PNI (aOR = 0.38, 95% CI: 0.22–0.65, P < 0.001) remained independent correlates of pancreatic cancer staging. Notably, the aOR for PNI was less than 1, indicating that a higher nutritional status index served as a protective factor for early-stage pancreatic cancer. Conversely, elevated levels of SII, SIRI, and CA19–9 were independently associated with an increased risk of advanced disease (stages III–IV). CEA also maintained statistical significance in the adjusted model (aOR = 1.56, P = 0.042). In contrast, age and gender did not show significant predictive value in either the univariate or multivariable models (all P > 0.05) (**Table 4**).

**Table 4:** Multivariable logistic regression analysis for early-stage vs. non-early-stage pancreatic cancer.

Variable	Unadjusted OR	95% CI (Unadj)	P value (Unadj)	Adjusted OR*	95% CI (Adj)	P value (Adj)
Age	1.02	0.98-1.06	0.32	1.01	0.97-1.05	0.58
Gender	1.08	0.65-1.79	0.76	0.95	0.54-1.67	0.85
CA19-9	3.45	2.12-5.62	<0.001	2.89	1.68-4.98	<0.001
CEA	1.89	1.12-3.18	0.018	1.56	0.89-2.74	0.042
SII	2.67	1.68-4.25	<0.001	2.12	1.28-3.51	0.003
SIRI	2.34	1.45-3.78	<0.001	1.78	1.05-3.02	0.032
PN	0.42	0.26-0.68	<0.001	0.38	0.22-0.65	<0.001

### 3.5 Discriminatory ability of individual biomarkers for pancreatic cancer stage

ROC curve analysis was performed to evaluate the ability of individual biomarkers to discriminate early-stage pancreatic cancer (**Table 5**). Among all single indicators, PNI demonstrated the best diagnostic performance, with an AUC of 0.78 (95% CI: 0.72–0.84). At the optimal cut-off value of 45.6, the sensitivity and specificity were 75.3% and 70.5%, respectively. SII and SIRI yielded AUC values of 0.72 (95% CI: 0.65–0.79) and 0.70 (95% CI: 0.63–0.77), respectively, indicating moderate diagnostic value. Among the conventional tumor markers, CA19–9 showed an AUC of 0.75 (95% CI: 0.68–0.81), whereas CEA exhibited a relatively lower diagnostic performance, with an AUC of 0.64 (95% CI: 0.57–0.71).

**Table 5:** Diagnostic performance of individual biomarkers for early-stage pancreatic cancer.

Indicators	AUC	95% CI	Cut-off	Sensitivity	Specificity
SII	0.72	0.65–0.79	823.4	68.40%	65.70%
SIRI	0.7	0.63–0.77	3.1	70.10%	63.20%
PNI	0.78	0.72–0.84	45.6	75.30%	70.50%
CA19-9	0.75	0.68–0.81	1000	71.20%	68.90%
CEA	0.64	0.57–0.71	8.1	62.50%	60.30%

### 3.6 Discriminatory ability of combined biomarker models for pancreatic cancer stage

The diagnostic performance of different combined models for early-stage pancreatic cancer was further compared (**Table 6**). Model A, which included CA19–9 alone, yielded an AUC of 0.75. In contrast, Model B, incorporating inflammatory and nutritional indices (SII + SIRI + PNI), showed improved diagnostic performance with an AUC of 0.82, and achieved a sensitivity of 78.9% and a specificity of 74.3%. Model C, which combined conventional tumor markers (CA19-9 + CEA), demonstrated an AUC of 0.77. The highest diagnostic performance was observed for Model D, which integrated all evaluated indicators (SII + SIRI + PNI + CA19-9 + CEA), with an AUC of 0.88 (95% CI: 0.83–0.92), a sensitivity of 84.6%, and a specificity of 81.2%. This performance was markedly superior to that of single biomarkers and partial combined models.

**Table 6:** Comparison of diagnostic performance among different models.

Model	Variable	AUC	95% CI	Sensitivity	Specificity
Model A	CA19-9	0.75	0.68–0.81	71.20%	68.90%
Model B	SII+SIRI+PNI	0.82	0.76–0.87	78.90%	74.30%
Model C	CA19-9+CEA	0.77	0.71–0.83	73.50%	70.10%
Model D	Combined	0.88	0.83–0.92	84.60%	81.20%

To ensure the reliability and stability of the diagnostic performance, internal validation was performed for the four predictive models (**Table 7**). Model A (CA19–9 alone) yielded an AUC of 0.71 (95% CI: 0.64–0.77), with a sensitivity of 68.6% and specificity of 69.8%. Model B, which integrated inflammatory and nutritional indices (SII+SIRI+PNI), showed an improved AUC of 0.78 (95% CI: 0.72–0.84). Model C (CA19-9 + CEA) demonstrated a diagnostic accuracy similar to Model A, with an AUC of 0.73. Notably, the fully integrated model, Model D (Combined SII, SIRI, PNI, CA19-9, and CEA), demonstrated the highest diagnostic efficacy in the internal validation, achieving an AUC of 0.84 (95% CI: 0.79–0.90). This model also exhibited superior clinical utility, with a sensitivity of 80.9% and a specificity of 82.6%. These validation results confirm that the multi-marker combination significantly enhances the identification of early-stage pancreatic cancer compared to any single marker or partial combination.

**Table 7:** Internal validation results for combined diagnostic model.

Model	Variable	AUC	95% CI	Sensitivity	Specificity
Model A	CA19-9	0.71	0.64-0.77	68.6%	69.8%
Model B	SII+SIRI+PNI	0.78	0.72-0.84	74.0%	76.30%
Model C	CA19-9+CEA	0.73	0.66-0.79	69.8%	71.5%
Model D	Combined	0.84	0.79–0.90	80.9%	82.6%

### 3.7 Supplementary analysis in CA19-9–negative patients

A subgroup analysis was further performed in patients with negative CA19–9 expression to evaluate the diagnostic value of inflammatory and nutritional indices (**Table 8**). The results showed that PNI maintained good diagnostic performance in this population, with an AUC of 0.79. The AUCs of SII and SIRI were 0.74 and 0.71, respectively. After combining inflammatory and nutritional indices, the diagnostic performance of the integrated model was further improved, achieving an AUC of 0.85 (95% CI: 0.78–0.91), with a sensitivity of 82.1% and a specificity of 79.3%. These findings indicate that inflammatory- and nutrition-related indices may serve as important complementary diagnostic tools for early-stage pancreatic cancer in CA19-9–negative patients.

**Table 8:** Diagnostic value of inflammatory and nutritional indices in CA19-9–negative patients.

Indicators	AUC	95% CI	Sensitivity	Specificity
SII	0.74	0.65–0.83	70.20%	68.50%
SIRI	0.71	0.62–0.80	68.90%	65.10%
PNI	0.79	0.71–0.87	76.40%	73.20%
Combined	0.85	0.78–0.91	82.10%	79.30%

## 4 Discussion

This study systematically evaluated the diagnostic value of inflammatory indices (SII, SIRI), nutritional status (PNI), and conventional tumor markers (CA19-9, CEA) for early-stage pancreatic cancer. The results demonstrated that although individual biomarkers possess moderate diagnostic capability, multi-marker combined models significantly improved both the sensitivity and specificity for early detection. In this section, we compare our findings with previous studies and discuss the significance of immune-inflammatory and nutritional status in the diagnosis and potential mechanisms of pancreatic cancer.

Our results indicated that among single biomarkers, PNI exhibited the highest diagnostic performance for early-stage pancreatic cancer (AUC = 0.78), followed by CA19-9, SII, and SIRI. The combination of inflammatory and nutritional indices further enhanced diagnostic efficacy, and the integrated model including all indicators reached an AUC of 0.88, highlighting the clinical value of comprehensive assessment of inflammation and nutritional status. In contrast, most previous studies have primarily focused on the prognostic applications of inflammatory and nutritional indices in cancer (11, 12). Teja M et al. (13) investigated preoperative nutritional and inflammatory indices in pancreatic cancer and reported that both PNI and SII were associated with postoperative recovery and long-term prognosis, with PNI showing more pronounced significance. This finding aligns with our observation that PNI is highly sensitive to disease-related indicators. Their study further demonstrated that PNI is closely correlated with overall survival (OS) and recurrence-free survival (RFS), while SII is also related to postoperative recovery. These findings support the notion that nutritional status reflects the biological behavior of pancreatic cancer and may serve as an important clinical indicator.

In terms of the value of systemic inflammatory indices, our study demonstrated that both SII and SIRI increased progressively with advancing pancreatic cancer stage and provided complementary diagnostic information when incorporated into combined models. Previous studies on SIRI have primarily focused on prognostic prediction rather than stage diagnosis (14, 15). Shen H et al. (16), through a meta-analysis of seven studies, found that elevated SIRI significantly predicted poorer OS and progression-free survival (PFS) in pancreatic cancer patients, indicating that SIRI is an important predictive biomarker. Similarly, another retrospective cohort study reported that SIRI was a stronger predictor of survival in pancreatic cancer patients than traditional markers such as the NLR and SII, highlighting its value as a long-term prognostic indicator (17). Although these studies primarily focused on prognosis, their findings are consistent with our observation of a positive correlation between SIRI and tumor stage and aggressiveness, suggesting that systemic inflammation not only contributes to disease progression but may also reflect early biological changes in tumors.

Comparable trends have been observed in other gastrointestinal malignancies, such as gastric cancer (18, 19). Jing et al. (20) demonstrated that in early-stage gastric cancer, both SII and PNI independently predicted patient prognosis, and the combined index model improved prognostic accuracy. This aligns with our finding that the combination of inflammatory and nutritional indices enhances diagnostic performance for pancreatic cancer, suggesting that these blood-based biomarkers may have diagnostic or prognostic applicability across different tumor types. Furthermore, comparisons with studies on other cancers strengthen the theoretical background of our study. For early-stage gastric cancer, a combined model incorporating CA19-9, SII, SIRI, and PNI demonstrated superior diagnostic performance compared with single markers, particularly in male patients (21). These findings support the potential advantage of multi-marker combined assessment for early tumor detection and underscore that integrating inflammatory and nutritional status can improve the diagnostic accuracy of conventional tumor markers. In addition, the prevalence imbalance between early-stage (59.1%) and non-early-stage (40.9%) patients in our cohort may have influenced the ROC performance. The higher proportion of early-stage patients could potentially lead to overestimation of sensitivity and underestimation of specificity due to spectrum bias, particularly given that our study was conducted at a referral center where early detection efforts may be more intensive. Future studies should aim for more balanced cohorts or apply correction methods such as stratified sampling or weighting approaches to validate the diagnostic performance of these combined markers.

At the mechanistic level, the relationship between inflammation and tumor development has been widely confirmed in both basic and clinical studies (22, 23). Chronic inflammation can promote genetic instability in pancreatic cells, the release of pro-inflammatory cytokines, and alterations in the tumor microenvironment, thereby facilitating tumor initiation and progression. Systemic inflammatory indices, such as SII and SIRI, reflect the dynamic balance of immune cells in peripheral blood, including granulocytes, lymphocytes, and monocytes. Their elevation may indicate a pro-tumor inflammatory microenvironment, which is consistent with previous studies showing that inflammation-mediated processes are closely involved in pancreatic cancer development (24). Moreover, poor nutritional status (low PNI) is often associated with impaired immune function, insufficient protein and energy reserves, and reduced immune surveillance against tumor cells, contributing to accelerated tumor progression.

To contextualize the clinical utility of our proposed multi-marker model, it is essential to compare its performance with existing clinical and radiologic diagnostic pathways for pancreatic cancer. Current standard practice for suspected pancreatic cancer typically involves a stepwise approach beginning with cross-sectional imaging, followed by endoscopic ultrasound (EUS) with tissue acquisition when indicated (25, 26). According to current guidelines, contrast-enhanced CT and MRI/MRCP serve as first-line imaging modalities, with diagnostic accuracies ranging from 86.5% to 98.0% for detecting established pancreatic lesions (27). However, these imaging techniques demonstrate significant limitations in detecting early-stage (stage I) pancreatic cancer, particularly for lesions smaller than 2 cm, with sensitivity dropping substantially for subcentimeter tumors (28). Endoscopic ultrasound (EUS) with fine-needle aspiration (FNA) or biopsy (FNB) represents the gold standard for tissue diagnosis and evaluation of small pancreatic lesions, offering superior sensitivity (91%-100%) compared to CT (53%-91%) (25, 27). Nevertheless, EUS is operator-dependent, invasive, and not suitable for population-wide screening.

The incremental value of our model becomes evident when considering the specific clinical scenario of CA19-9-negative patients, who constitute 5%-10% of the pancreatic cancer population (4). In this subgroup, conventional tumor marker screening fails completely, whereas our combined inflammatory-nutritional model maintained robust diagnostic performance (AUC = 0.85). Furthermore, recent economic evaluations have highlighted that biomarker-based screening strategies, when sequentially applied to enrich high-risk populations (such as those with new-onset diabetes), may approach cost-effectiveness thresholds by potentially reducing the number of unnecessary imaging procedures (29). In our study, the full combined model demonstrated superior diagnostic accuracy compared to CA19–9 alone (AUC 0.88 vs. 0.75), suggesting potential incremental value that warrants further investigation regarding its role in optimizing imaging referral pathways. However, we acknowledge that these findings are preliminary and hypothesis-generating; prospective studies are needed to validate whether such a multi-marker strategy can effectively reduce unnecessary imaging in clinical practice.

The clinical significance of this study lies in proposing a multidimensional combined strategy based on routine peripheral blood tests for stage diagnosis of pancreatic cancer, particularly providing complementary diagnostic value for CA19-9–negative patients. However, several limitations exist. First, this was a single-center retrospective study, which may introduce selection bias and limit the generalizability of our findings to broader populations. These methodological constraints raise concerns about potential overfitting, particularly given the single-center retrospective design and the moderate sample size. Consequently, the reported AUC values for the combined models may represent optimistic estimates of diagnostic performance. Future studies should employ rigorous statistical modeling with appropriate validation procedures to confirm our findings and establish reliable cut-off values for clinical application. Second, inflammatory and nutritional indices can be influenced by various non-tumor factors (e.g., infections, chronic diseases), potentially affecting their specificity. Finally, larger-scale, multicenter prospective studies are needed to validate the generalizability of the combined model developed in this study.

In summary, our findings are consistent with previous research regarding the role of inflammatory and nutritional indices in tumor diagnosis and prognostic evaluation, further supporting the potential value of SII, SIRI, and PNI in clinical tumor assessment and introducing a novel strategy for their combined use in early-stage pancreatic cancer detection.

## 5 Conclusion

This study systematically evaluated the clinical value of SII, SIRI, PNI, and conventional tumor markers (CA19-9, CEA) for the stage diagnosis of pancreatic cancer. The results indicate that enhanced systemic inflammation, deteriorated nutritional status, and elevated tumor markers are closely associated with disease stage progression and lower tumor differentiation, suggesting that immune–inflammatory dysregulation and malnutrition play important roles in pancreatic cancer development. The combined assessment of systemic immune–inflammation indices, nutritional status, and tumor markers provides an effective and broadly applicable auxiliary tool for early identification of pancreatic cancer. This approach has the potential to inform clinical early screening, risk stratification, and individualized management. Future multicenter, prospective studies are warranted to further validate its stability and clinical utility.

## Data Availability

The original contributions presented in the study are included in the article/supplementary material. Further inquiries can be directed to the corresponding authors.
